# Erratum for Ciccone et al., “Rapid Diagnostic Tests to Guide Case Management of and Improve Antibiotic Stewardship for Pediatric Acute Respiratory Illnesses in Resource-Constrained Settings: a Prospective Cohort Study in Southwestern Uganda”

**DOI:** 10.1128/spectrum.00443-22

**Published:** 2022-02-23

**Authors:** Emily J. Ciccone, Lydia Kabugho, Emmanuel Baguma, Rabbison Muhindo, Jonathan J. Juliano, Edgar Mulogo, Ross M. Boyce

**Affiliations:** a Division of Infectious Diseases, University of North Carolina School of Medicine, Chapel Hill, North Carolina, USA; b Department of Community Health, Mbarara University of Science and Technology, Mbarara, Uganda; c Department of Epidemiology, Gillings School of Global Public Health, Chapel Hill, North Carolina, USA

## ERRATUM

Volume 9, no. 3, e01694-21, 2021, https://doi.org/10.1128/Spectrum.01694-21. Page 6, line 6 “88%” should read “92%.”

Page 7, Fig. 3 legend, line 5, “<0.5” should read “>0.5.”

